# Our Quarter Centennial

**Published:** 1878-12

**Authors:** 


### HALL'S JOURNAL OF HEALTH.

E. H. GIBBS, A.M., M.D., Editor,

We aim to show how Disease may be Avoided.

Vol. XXV.] December, 1878. [No. 12

### *OUR QUARTER-CENTENNIAL.*

With this number, Hall's Journal of Health completes its Twenty-fifth Year. For three hundred consecutive months it has labored to instruct men how to live to secure health, happiness, and long life. It seems but a little while to look back to the date of the first number. Yet, since that time, more than three-quarters of the people then living have passed away, and others have taken their places. And we know that the next quarter century will be just as replete with the lights and shadows that make up the sum of human life, and at its close, the majority will in their turn have perished from the earth. In view of these facts, how trivial are the cares, anxieties, and even afflictions of those who trustingly look upward. All that the world can give is not enough to insure one hour of real happiness.

" For ghosts unseen creep in between,
And when our songs flow free,
Sing discords in an undertone,
And mar the harmony."

Hall's Journal of Health was the first Health Journal ever published, and it is now the only one. Its principal support comes from the intelligent and cultivated classes who can best appreciate its teachings. Newspapers quote from it more extensively than from any other publication. Next month Hall's Journal of Health will enter upon its twenty-sixth year, with the prestige of a wide popularity, and the best wishes of every philanthropist. We have not the wisdom to forecast its future, but we believe it will continue to live and to prosper after this generation shall have passed away.

Hall's Journal of Health is published

| | |
|---|---|
| Monthly at | $1.50 a year |
| Eight months | 1.00 |
| Two copies one year | 2.75 |
| Three copies " " | 4.00 |
| Four copies " " | 5.00 |
| Each additional copy | 1.25 |
| To News Agents—Booksellers—Physicians—Clergymen —Benevolent and Religious Societies—and Public Libraries | 1.00 a year |

As each number is complete in itself, subscriptions may commence at any time. Send fractions of a dollar in postage stamps. Address E. H. Gibbs, M.D., Editor Hall's Journal of Health, New York.

